# Ethics of artificial intelligence in embryo assessment: mapping the terrain

**DOI:** 10.1093/humrep/deae264

**Published:** 2024-12-09

**Authors:** Julian J Koplin, Molly Johnston, Amy N S Webb, Andrea Whittaker, Catherine Mills

**Affiliations:** Monash Bioethics Centre, Monash University, Clayton, VIC, Australia; Monash Bioethics Centre, Monash University, Clayton, VIC, Australia; Monash Bioethics Centre, Monash University, Clayton, VIC, Australia; School of Social Sciences, Monash University, Clayton, VIC, Australia; School of Social Sciences, Monash University, Clayton, VIC, Australia; Monash Bioethics Centre, Monash University, Clayton, VIC, Australia

**Keywords:** artificial intelligence, embryo selection, embryo assessment, AI transparency, bioethics, machine learning

## Abstract

Artificial intelligence (AI) has the potential to standardize and automate important aspects of fertility treatment, improving clinical outcomes. One promising application of AI in the fertility clinic is the use of machine learning (ML) tools to assess embryos for transfer. The successful clinical implementation of these tools in ways that do not erode consumer trust requires an awareness of the ethical issues that these technologies raise, and the development of strategies to manage any ethical concerns. However, to date, there has been little published literature on the ethics of using ML in embryo assessment. This mini-review contributes to this nascent area of discussion by surveying the key ethical concerns raised by ML technologies in healthcare and medicine more generally, and identifying which are germane to the use of ML in the assessment of embryos. We report concerns about the ‘dehumanization’ of human reproduction, algorithmic bias, responsibility, transparency and explainability, deskilling, and justice.

## Graphical Abstract

**Figure:**
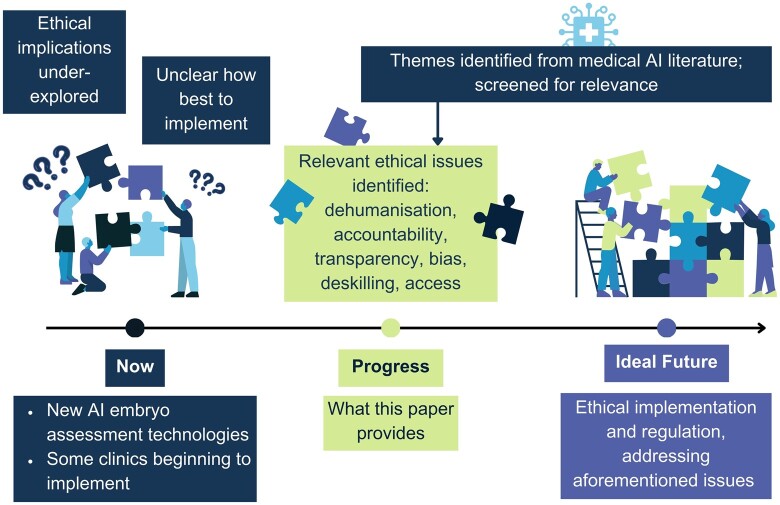
**While artificial intelligence may offer clinical benefits for embryo assessment, its successful implementation requires awareness of the ethical issues that the technology raises.** AI, artificial intelligence.

## Introduction

ARTs provide many people with their best chance of achieving a goal to which they ascribe great importance: parenthood. However, it has never been a straightforward method. The financial expense of ART is significant (estimated at $8000–$15 000 USD per non-donor cycle in the USA; Thompson, 2016). Undergoing fertility treatment and experiencing failed ART cycles can have a significant emotional toll (Cousineau and Domar, 2007; Verhaak *et al.*, 2007). Interventions that can decrease the time to pregnancy thus have important clinical and moral considerations in their favour.

Machine learning (ML) tools (a type of artificial intelligence (AI)) to improve the selection of human embryos for transfer (Luong and Le, 2024) may be one such intervention. Promising results have been published in high-profile scientific journals (Riegler *et al.*, 2021; Delestro *et al.*, 2022; Dimitriadis *et al.*, 2022), and some ML tools have already reached the market (Life Whisperer Diagnostics, n.d.; Merck Group, 2023; Vitrolife, 2023).

ML embryo assessment tools work by analysing time-lapse images and/or videos of embryos at various stages of development, and then providing predictions of embryo quality based on the embryo’s morphology and morphokinetics. These tools are currently used in conjunction with embryologist grading but also have the potential to replace human grading altogether. Early evidence suggests that ML tools can outperform human embryologists, produce more consistent embryo assessments (Riegler *et al.*, 2021), and significantly reduce assessment times (Illingworth *et al.*, 2024).

Given the clinical promise of ML embryo assessment tools and their recent integration into many clinics, there is an urgent need to determine how best to implement this technology. A deep understanding of the relevant ethical, social, and regulatory issues is necessary to ensure that this technology is used in a way that is ethically appropriate and preserves public trust in the field of ART. Few AI technologies touch on anything as intimate as human reproduction, or deal with material with as much moral significance as human embryos. It is therefore crucial to identify the ethical issues related to the use of AI in this space—both general AI issues and those raised by the specific, morally fraught context of human reproduction. Managing these ethical issues appropriately will be crucial to preserving trust in both ML embryo assessment specifically, and ART more generally.

So far the ethical questions raised by ML embryo assessment have received negligible scholarly discussion. This article contributes to this nascent area of research by surveying the key ethical concerns raised by ML technologies in general (with a particular focus on medical AI) and by identifying which are germane to the use of ML for embryo assessment. Our selection of ethical issues represents major topics in AI ethics guidelines and cluster largely around accountability, transparency, justice/fairness, and respect for autonomy (Floridi and Cowls, 2019; Jobin *et al.*, 2019; Hagendorff, 2020). We acknowledge that such lists of principles have been described as under-specified and/or incomplete (Jobin *et al.*, 2019; Hagendorff, 2020; Munn, 2023). Accordingly, we supplement our analysis with a survey of the applied ethics literature on other AI technologies that raise overlapping issues relevant to ML embryo assessment. We assume throughout that ML embryo assessment tools will be accurate, consistent, and clinically useful, but note that further research may be necessary before ML embryo assessment can be said to have fully met this threshold (Kragh *et al.*, 2021; Sfontouris *et al.*, 2022). We acknowledge that in the absence of genuine benefits, selling ML embryo assessment to patients would be premature and arguably exploitative.

## Dehumanization

One major worry about AI is that its use can be *dehumanizing.* As the term ‘dehumanization’ suggests, the concern here is that computer algorithms can fail to treat us with the kind of consideration that human beings deserve. Susskind (2022) provides a characteristic expression of this worry:

‘… to treat people as mere data points in a larger algorithmic exercise is to risk violating the principle that *every person counts.* When we stand before a judge, an employer, a parole officer, or a mortgage lender, we want to be seen for who *we* really are, rather than as mere bundles of shared traits.’

Dehumanization worries have also found purchase in the medical domain, where many consumers worry that reducing patients to a number can miss important characteristics of them. This concern—sometimes described in terms of ‘uniqueness neglect’ (Longoni *et al.*, 2019)—likewise reflects a belief that algorithms can fail to treat us with the full measure of respectful consideration that medicine requires.

In applying dehumanization worries to ML embryo assessment, we first need to ask who stands to be ‘dehumanized’ by the process: the patient or the embryo? While it might be disrespectful or otherwise unethical to roll out ML embryo assessment without consulting patients’ preferences (a possibility we discuss further below), the use of the technology does not inherently treat *patients* as less than fully human. Indeed, it makes no judgements about patients whatsoever; it is patients’ *embryos* that are assessed.

Can embryos themselves be ‘dehumanized’ through the AI selection process? We argue not. While human embryos are in some biological sense human, the respect with which we are generally expected to treat embryonic life is limited. Even those opposed to abortion usually do not view spontaneous miscarriage in the early weeks of embryonic life as a moral tragedy akin to the death of an adult (Ord, 2008). As many bioethicists have argued, the attitudes we take towards human adults need not extend to human embryos, any more than the attitudes we take towards chickens extend towards fertilized chicken eggs (Williams, 1985). Indeed, many philosophers argue that it is not our humanness but our ‘moral personhood’, in the sense of some set of capacities like rational thought, self-consciousness, and/or sentience, that makes us worthy of significant respect (Bortolotti and Harris, 2005; Rowlands, 2016; Andrews *et al.*, 2018). Hence, the kind of respect due to human persons need not extend to human embryos.

Nonetheless, it is sometimes argued that despite lacking moral status of the sort that human persons have, human embryos have a kind of moral or symbolic value, and hence ought to be treated respectfully (Sandel, 2004). We should therefore ask whether ML embryo assessment might fail to treat embryos with the respect that some philosophers believe they deserve. However, it is not clear that human health professionals treat embryos with greater respect than an AI system would. Both human and AI decisions about which embryo to transfer are driven by visual features of the embryo that can be detected under the microscope. To make major decisions about a human person based merely on their visual features *would* be disrespectful—but this process is unavoidable in the context of embryo selection, whether performed by a human or AI.

## Bias

The risk that AI systems can display unintended or unanticipated forms of bias is now widely understood (O’Neil, 2017; Jobin *et al.*, 2019). This bias can take various forms. The first possibility is that ML algorithms will perform better for members of some groups than for others (e.g. based on ethnicity), potentially reflecting differences in how well these groups are represented in the datasets on which AI algorithms are trained. Such performance gaps are a recognized problem within medical AI (Obermeyer *et al.*, 2019) and, as we explain below, there is reason to think they may likewise be relevant to ML embryo assessment (Afnan *et al.*, 2021).

Performance gaps in ML embryo assessment (and how well it performs for different demographics) are clearly undesirable. However, precisely what should be done when an algorithm performs better for members of one group than another is less clear. An ideal solution would be to improve the algorithm’s performance for worse-performing groups, potentially by retraining it on more representative data. Unfortunately, this solution will not always be available since it depends on access to data that may simply not exist.

An alternative response involves implementing what are known as ‘fairness algorithms’ to equalize performance between better-performing and worse-performing groups. However, algorithmic solutions to unequal performance can risk worsening performance for better-performing groups *without* improving performance for the worse-performing groups. Some AI ethicists have argued that obtaining equality purely by worsening the position of the well-off does not improve matters (Mittelstadt *et al.*, 2023). If equal performance of ML embryo assessment tools cannot reasonably be achieved in other ways, it will become necessary to consider whether the implementation of ‘fairness algorithms’ of the kind discussed here would be a moral improvement, or whether unequal performance should instead be tolerated.

Alternatively, ML embryo assessment tools might display a distinct form of ‘bias’ if their outputs track features that are irrelevant to the chance of ART success—e.g. if they look for particular embryo features that embryologists have historically favoured (which may or may not be truly beneficial.) It may also take into account features that patients would not want to influence the choice of embryo (e.g. if the AI system is more likely to recommend transferring embryos of a particular sex, or, theoretically, embryos with disease traits that happen to correlate with a higher chance of implantation; Afnan *et al.*, 2021).

While intuitively concerning, bias in this second sense is not unique to AI systems. Despite following a consistent grading system, assessment of embryo quality varies between clinics and between embryologists depending (inter alia) on where they have trained, by whom they were trained, and their clinical experiences to date (Storr *et al.*, 2017). This variability arguably also reflects ‘bias’ insofar as different approaches to embryo selection likewise take irrelevant and/or undesirable features into account. Whether AI bias presents different issues to (or more serious issues than) human bias is thus an open question.

## Responsibility

One recurring worry about ML tools is that they can open ‘responsibility gaps’, whereby it becomes impossible to rightly hold any human agent responsible for adverse outcomes (Matthias, 2004). Since ML tools largely ‘teach themselves’ how to perform particular tasks, they are not fully in the control of either the programmer/manufacturer *or* the human operator/supervisor. An ML tool may thus fail to perform as expected in ways that could not have been reasonably foreseen by anyone involved (Rudy-Hiller, 2022). The problem is that it is commonly thought that agents should only be held responsible for outcomes that are in their control. This is not always the case for ML tools, which can make responsibility for undesirable outcomes caused by such tools difficult to trace. This raises the question of who (if anyone) should be held responsible if the use of ML embryo assessment tools unexpectedly *increases* average time to pregnancy or reduces live birth rates (e.g. if viable embryos are discarded.)

Similar questions arise for ML tools designed to predict embryo ploidy status (Bamford *et al.*, 2023; Jiang and Bormann, 2023). In this case, it is unclear who (if anyone) ought to be held responsible if the ML tool provides a false negative, or an embryo is selected for transfer, and the pregnancy results either in miscarriage or the birth of a child with an aneuploidy. This latter possibility raises a philosophical quandary known as the ‘non-identity problem.’ The question here is whether wrongdoing has occurred if a child is created with a lower expected quality of life than a hypothetical different child. While some hold the intuitively appealing view that wrongdoing has occurred in such situations, there is a philosophical difficulty here: it is not clear how a child could be harmed or wronged by being born, provided that their life is at least good enough to be worth living, and their birth thus benefits them (Brock, 1995; Boonin, 2008). While there is controversy around whether ‘life worth living’ is an appropriate standard to apply, further exploration of the non-identity problem for reproductive technologies is beyond the scope of this article.

Leaving the non-identity problem aside, it seems clear that ML embryo assessment tools could leave prospective parents worse off if they fail to perform as expected. Assigning responsibility for such negative outcomes is complicated. It might be thought that we can leave current lines of responsibility intact by using ML tools purely as a decision aid, with ultimate decision-making responsibility lying with human embryologists. However, this measure would not necessarily ensure embryologists carefully interrogate ML outputs: social psychological research into ‘automation bias’ suggests that human decision-makers often place excessive weight on information provided by automated decision-making systems (Lyell and Coiera, 2017). More importantly, if an AI consistently outperforms human experts, then these experts could be argued to have an *obligation* to defer to the AI decision-making system, much as they ought to defer to a human expert whose judgement is consistently more reliable than their own. If it is inappropriate to override the recommendations of an AI system that outperforms you, then it would be a ‘decision aid’ in name but not in practice. To continue to hold embryologists responsible for decisions *for which they have a moral obligation to defer to AI* seems perverse.

In addition to backwards-looking responsibility for negative outcomes, it has been argued that collective forward-looking responsibilities may be one way to tackle the responsibility gap and ensure medical AI is implemented ethically (Ferlito *et al.*, 2024). On this view, responsibility would be collectively shared among relevant stakeholders. Applying this approach to ML embryo assessment would require the identification of relevant stakeholders and collective deliberation regarding the full scope of potential issues that may arise from the use of this technology, which is outside the scope of this review.

There are various objections to the idea that responsibility gaps pose a genuine problem for the implementation of new AI technologies. Some theorists hold that current approaches to responsibility ascription, carefully applied, can locate responsibility for poor outcomes of AI technologies (Tigard, 2021). Even insofar as AI systems can negate or erode responsibility, it might be the case that we are sometimes *overly* prone to hold others responsible for regrettable outcomes (Danaher, 2022; Munch *et al.*, 2023). Perhaps many of our practices involve excessive responsibilization, and, accordingly, some responsibility gaps should be welcomed.

Even if responsibility gaps *are* both unavoidable and undesirable, their downsides might be an acceptable price to pay for improved overall performance. If ML tools significantly outperform (unaided) human embryologists, the benefits to patients might trump the importance of being able to trace responsibility for the rare undesirable outcomes that still occur. Whether somebody can be held responsible for poor outcomes might be less important than the overall balance of harms and benefits.

Finally, it is worth noting that responsibility gaps are often considered most acutely problematic in cases of life-and-death decision-making. For example, the UN Principles for the Ethical Use of Artificial Intelligence hold that life and death decisions must not be ceded to machines (United Nations Systems Chief Executives Board for Coordination, 2022). One question for ML embryo assessment is whether decisions about which embryo is transferred fall within the scope of a ‘life or death decision’ for which responsibility gaps are often considered unacceptable. Decisions about which embryo will be given a chance of life are, in a sense, decisions about ‘life or death’—but about the creation of life, not the possible deaths of existing persons. The significance of moral responsibility for decisions about *creating* life remains an open question.

## Deskilling

Medical AI, if it functions well, can improve clinical decision-making. Where AI outperforms humans, it might be understandable if clinicians come to rely on, and defer to, AI systems. This might lead to a deterioration of clinical skills via a phenomenon often described as ‘deskilling’ (Lu, 2016; Duran, 2021).

Deskilling worries are particularly pertinent to ML embryo assessment, since there are good reasons to think ML assessment tools will not be taken up equally across the industry (inter alia, because not all clinics have access to the necessary time-lapse imaging.) Any skill deterioration could lead to worse outcomes for patients if embryologists who have come to rely on ML tools go on to work in contexts where they must assess embryos manually. Skill maintenance will also be an important safeguard if the technology fails (e.g. via ‘catastrophic forgetting’, adversarial attacks, data poisoning, or other potential vulnerabilities of ML models; Hatherley and Sparrow, 2023). Deskilling might prevent embryologists from performing their role effectively if they are required to review or provide oversight of ML recommendations, or if ultimate decision-making responsibility falls on them.

Avoiding deskilling will also be important for the management of other ethical concerns. For example, one strategy to manage worries about AI bias is to routinely check the performance of the algorithm. However, embryologists cannot perform this role effectively unless they maintain manual assessment skills.

## Transparency and explainability

In both AI ethics and computer sciences research, the terms ‘transparency’, ‘interpretability’, and ‘explainability’ are used in varying and sometimes contradictory ways (Lipton, 2018; Cortese *et al.*, 2023). We can see two main sets of concerns regarding transparency in AI ethics. One relates to whether one can understand why an AI algorithm has made a particular decision. The other relates to whether one is aware that AI has been used. Both worries are driven by opposition to important decisions about our lives being made in ways that we do not understand. However, the kinds of transparency at stake—and what is required to achieve it—differ between these two cases. We therefore consider them separately.

### Transparency and explainability of ML embryo assessment tools

ML tools can process vast amounts of data and make predictions or decisions based on patterns they identify. However, these tools often operate as ‘black boxes’, meaning that while we can observe their input and output, the internal mechanisms driving their decisions are not easily interpretable by humans (Luong and Le, 2024). Most AI models in computer vision (the field of AI on which embryo assessment models are built) are ‘black boxes’; hence, their internal reasoning processes are not easily understood by the human users or designers of these systems (Rudin, 2019).

Opaque or ‘black box’ AI is widely considered unacceptable for medical contexts. Muller and colleague’s ‘Commandments of Ethical Medical AI’ holds that ‘AI decisions, actions, and communicative processes must be transparent and explainable’ (Muller *et al.*, 2021). Bjerring and Busch (2021) hold that black box systems are incompatible with informed consent, since they prevent clinicians from ‘present[ing] the [relevant medical] information in a way that enables a patient to comprehend and process it rationally’. These reservations about the use of black box medical AI models plausibly extend to ML embryo assessment, as the internal reasonings behind the ML outputs are not interpretable and hence are unexplainable by those overseeing the technology.

There are two distinct ways of addressing the black box problem (Lipton, 2018; Mittelstadt, 2022). The first—sometimes termed ‘transparency’ or ‘intrinsic interpretability’—involves limiting model complexity so that the user is able to understand how the model works. Indeed, the limited existing ethical analyses of embryo assessment recommend building interpretable AI for this purpose (Afnan *et al.*, 2021; Luong and Le, 2024). However, prioritizing transparency may come at the expense of performance, as opaque models can be more complex and have a greater capacity for higher performance than transparent models (London, 2019; Wang *et al.*, 2020). The second approach to the black box problem—sometimes described as ‘*post hoc* interpretability’—involves finding some way to interpret an opaque AI model after the fact, e.g. by building a simpler, transparent model that approximates the behaviour of the black box model (and might thereby indicate roughly how the more complex model functions.) Unlike building a transparent (or ‘intrinsically interpretable’) model, this does not require model complexity to be sacrificed. However, *post hoc* interpretations do not truly explain what is happening in the model; it is therefore an open question whether this approach is a true solution to the problem of AI opacity (Hatherley *et al.*, 2022), and thus whether those concerned with explainability in ML embryo assessment ought to insist on transparent/intrinsically interpretable systems.

At the same time, the case for interpretable medical AI faces criticisms of its own. The first holds that insistence on interpretability involves a double standard with respect to the degrees of transparency we accept from human decision-makers. Human decision-making is itself opaque; we often cannot or do not fully explain our decisions to others, and indeed frequently rationalize our decisions *to ourselves* after the fact. The opacity of human decision-making raises the question of why we should expect greater transparency from AI decision-makers than human ones (Zerilli *et al.*, 2019). While there appears to be no conclusive response in the literature yet, model transparency may be *instrumentally* valuable in properly allocating responsibility in instances when AI does not perform as intended (see discussion of responsibility earlier in the paper).

The second criticism argues that the importance of transparency (and explainability) has been overstated. Alex John London, for example, argues that accuracy should be our main priority (London, 2019). One reason is because any reductions to accuracy will lead to worse patient outcomes. Another is that medical ‘knowledge’ is itself opaque and associationist in a similar way to AI judgements. For example, lithium is commonly prescribed as a mood stabilizer even though we do not currently understand its mechanism of effect. London draws on this analogy to argue that what matters is whether an intervention is *effective*, not whether we fully understand how it operates. He describes a blanket preference for transparent models over opaque ones (even when the opaque models are more accurate) as a ‘lethal prejudice’ (London, 2019).

It is worth noting that *accuracy* is not, by itself, enough to guarantee better clinical outcomes. If ‘black box’ AI is distrusted by clinicians and/or patients, it might not be as widely accepted or deployed as a transparent (albeit less accurate) model might be. As Hatherley *et al.* (2022) point out, differences in uptake might counteract or outweigh any benefits from greater accuracy. It is thus not accuracy *per se* but instead clinical utility that needs to be considered.

Finally, it is worth noting that some argue that interpretable models are not always less effective than opaque ones (Rudin, 2019). If interpretable ML embryo assessment models are developed and can equal or surpass the performance of ‘black box’ models, then the former should be preferred over the latter insofar as (as some argue) transparency can arguably promote patient autonomy (by making the reasons behind recommendations clearer), non-maleficence (by facilitating error detection), and justice (by facilitating detection of bias) (Segers and De Proost, 2024). By contrast, opacity is not in and of itself a virtue. While black box AI might be justifiable when it has greater clinical utility, there are grounds to prefer transparent models when black box and transparent systems would be equally or similarly effective.

### Transparency regarding the use of ML embryo assessment

So far, we have been discussing the transparency *of* ML embryo assessment tools to ART providers. However, ART providers are not the only relevant stakeholders. Patients, too, may have an interest in understanding the technologies that are used in their treatment, and potentially in deciding for themselves whether they are comfortable with their use.

This raises the question of whether patients ethically ought to be informed of, and even explicitly consent to, the use of ML embryo assessment tools. (We bracket off questions about whether this disclosure would be *legally* required but note that in many jurisdictions it appears that disclosure of the use of medical AI is not always required; Cohen, 2019). This is a pressing question, since clinics are already beginning to implement such tools into their practice—and it is unclear how many of these clinics disclose the use of ML tools or provide patients with an opportunity to opt out.

There are some reasons why disclosure might seem unnecessary. The use of ML embryo assessment tools might be thought not to fundamentally change the nature or risks of assisted reproduction treatments. Whether performed manually by an embryologist or augmented by an AI model, embryo selection remains a process that patients have already consented to when they choose to undergo fertility treatment. It might seem that the use of AI does not change the stakes in any way that would be material to patients, particularly if the ultimate decision about which embryo to transfer is still made by embryologists. Indeed, knowledge of every tool used by a professional isn’t normally expected in other professions, and even in relation to healthcare it is sometimes argued that patients are not owed information regarding any AI tools informing their doctors’ clinical recommendations (Dunn and Nan, 2023). From this perspective, AI is one tool among many that can inform a medical professional’s recommendations, and does not need to be disclosed any more than discussions with colleagues that have likewise informed the professionals’ judgement.

Yet some patients may have serious objections to the use of medical AI that do not apply to other sources of information. Notably, some philosophers have argued that patients should have a right to withdraw from AI diagnostics and medical treatment planning, given possible reservations about AI bias, opacity, and possible long-term societal effects (Ploug and Holm, 2020). While AI embryo assessment is a distinct domain, similar considerations apply. Indeed, patients’ objections to the use of AI may be particularly acute when the domain in which it would be employed is as sensitive as human reproduction.

It is debatable whether patients’ reservations about the use of AI are well-founded. But the rationality of these objections is arguably beside the point; we should respect the values of patients, regardless of whether these values are ones that we personally share or even see as rationally justified. This principle is largely accepted even in relation to life-saving medical interventions—e.g. where mentally competent Jehovah’s Witnesses refuse life-saving blood transfusions (Bock, 2012). If patients have a strong objection to the use of ML embryo assessment, we can see no good reasons against accepting their judgement here.

It is worth noting, however, that there may be instances where embryo assessment cannot be performed without ML tools (e.g. where there is no available embryologist to perform manual assessment). In such instances, the options available to patients who object to the use of ML would be limited and the choice to opt-out of ML embryo assessment may not be possible without pursuing treatment elsewhere. It is thus important to consider whether clinics are morally obliged to maintain the option of manual human assessment for their patients.

## Access

Like other novel healthcare interventions, ML embryo assessment tools raise questions of access. These tools are not cost-free. Yet if they significantly improve treatment outcomes, whether or not one is able to access them will affect one’s odds of achieving this highly desired outcome. Many countries offer at least some level of public funding for fertility treatment. There is thus precedent for thinking that access to *effective* treatments is a matter of justice—and that interventions that improve the odds of a successful outcome or reduce time to pregnancy should potentially likewise be publicly funded or subsidized. One key question, then, is what (if any) measures should be taken to make ML embryo assessment tools readily accessible to all people undergoing assisted reproduction. For example, one might ask whether governments should support access to ML embryo assessment tools.

These questions are not wholly unique to ML embryo assessment. ART clinics already offer a range of optional (paid) ‘add-ons’ to patients, such as endometrial scratching, pre-implantation genetic testing for aneuploidy, and time-lapse imaging. There is a potential but notable similarity between ML assessment tools and adds-ons. Randomized control trials have found no, weak, or conflicting evidence that existing add-ons improve the chances of having a baby through assisted reproduction (Human Fertilisation and Embryology Authority, n.d.), which undermines the idea that access to these interventions is a matter of justice. If this turns out to also be true of ML embryo assessment, then access may not be an issue of justice but rather raise other issues such as exploitation. For instance, if clinics unduly promote or even overstate the benefits of ML embryo assessment, patients may be nudged into seeking—and possibly paying extra for—a technology with unproven benefits. There are, however, some grounds to hope that ML embryo assessment tools will eventually yield significant validatable benefits even if other add-ons do not. If this hope is realized, ML embryo assessment may deserve special ethical attention.

## Conclusion

Despite potential clinical advantages, such as decreased time to pregnancy and decreased miscarriage rates, ML embryo assessment raises significant ethical concerns that demand careful attention. These include worries about dehumanization, bias, responsibility gaps, deskilling, transparency, and equitable access.

These ethical concerns do not amount to arguments against the use of ML tools in embryo assessment. However, they do highlight that ethical implementation requires attending carefully to such concerns, mitigating them where possible, and making deliberate, considered decisions about how accountability for clinical outcomes will be managed, how the performance of ML tools will be monitored, and how their use will be communicated to patients. Ongoing ethical scrutiny, ideally involving input from philosophers, professionals, patients, and members of the public (Savulescu *et al.*, 2021), will be essential for harnessing the benefits of AI in reproductive medicine responsibly.

## Data Availability

Data availability does not apply to this article as no new data was created for this review.
